# 

**Published:** 1899-03-25

**Authors:** 


**IMPORTANT NOTICE.**

**On account of the Easter Holidays, we beg to announce that we shall go to press with our issue of the 1st APRIL on TUESDAY NIGHT, the 28th INST. Advertisements intended for insertion in this issue should therefore reach us BY NOON ON TUESDAY, the 28th inst.**

**Notices of Births, Deaths, and Marriages** are inserted in this column at a charge of 2s. 6d. for an announcement not exceeding four lines.

### NOTICE TO CORRESPONDENTS.

All MS., Letters, Books for Review, and other matters intended for the Editor should be addressed The Editor, "The Hospital," The Hospital Building, 28 & 29, Southampton Street, Strand, W.C.

The Editor cannot undertake to return rejected MS., even when accompanied by stamped directed envelope.

**Notice to Advertisers and Subscribers.**

All Advertisements, Orders for Copies of The Hospital, and Business Communications should be addressed to **THE MANAGER (Not to the Editor),** The Hospital, The Hospital Building, 28 & 29, Southampton Street, Strand, London, W.C.

The Cost of Subscription, post free, is as follows:—Per week, 2½d.; per quarter, 2s. 8d.; per half-year, 5s. 3d.; per annum, 10s. 6d. Foreign:—Per annum, 15s. 2d., payable, in all cases, in advance.

**NOTICE.—Vol. XXIV. of "The Hospital" (April, 1898, to September, 1898), in handsome cloth binding, is now on sale at the Office of this Journal, price 7s. 6d. Cases for binding the Numbers contained in this Volume 1s. 6d. Index to Vol. XXIV., 2½d., post free.**

**The Monthly Part for March is now ready. Price 6d., by post 8d.**

Influenza has been very prevalent during the past week in London. Deaths have been very numerous from pulmonary affections following the attacks.

### SCRAPS AND GLEANINGS.

A legacy of £100 has been left under the will of Mr. Francis Richards to the Hereford Infirmary.

The executors of the late Mr. E. Gannon have, out of the funds left by that gentleman, given £200 to the funds of the Hanwell Cottage Hospital building fund.

The British Home for Incurables, Streatham, has received contributions from the following City Companies: Clothworkers, 20 guineas; Leathersellers, 20 guineas; Dyers, three guineas.

H.H. the Maharani Saheb Gaekwar, of Baroda, paid a visit recently to the Cama and Allbless Hospitals, and has since generously forwarded a donation of Rs. 200 towards the endowment fund.

The Duchess of York will visit the Lynn and West Norfolk Hospital in April to assist in clearing off the debt incurred in commemorating the Queen's Diamond Jubilee by extending and improving the hospital.

A new infirmary was recently opened in connexion with the union workhouse buildings at Ripon. The cost of the building, exclusive of furnishing, has been about £2,750. It provides accommodation for 38 patients.

New York Red Cross Hospital, formerly in West 100th Street, which was closed during the summer and fall owing to the absence of Dr. and Mrs. Lesser and the Red Cross Sisters at the front, has been reopened at No. 259, West 93rd Street.

The workmen employed at the Old Park Works, Wednesbury, belonging to the Patent Shaft Company, have subscribed £5 5s. to the Wednesbury Nurses' Institute, £35 to the West Bromwich and District Hospital, and £16 to the Birmingham hospitals.

At a meeting of the Bulawayo Hospital Board, held on January 10th, a letter from Mr. K. B. Glosg, hon. secretary to Grey's Scouts, was read, handing over to the board the memorial gates erected at the hospital by the Scouts in memory of their late comrades.

The ladies of the Hospital Co-operating Association have handed over a sum of £364 12s. which they have collected during the last year on behalf of the Mater Hospital, Crumlin Road. This brings up the total which has been collected for this hospital by the members of this association to £2,000.

Messrs. Shakespear, Kirland, and Frost, Birmingham, recently gave a bicycle to be ballotted for at one penny per ticket, the proceeds to be handed over to the Children's Hospital. We understand that a cheque for 100 guineas has already been handed over to the hospital, that a little more is yet expected, and that the holder of the lucky ticket is a nurse at one of the local hospitals.

A poem, eulogistic of Sir Squire Bancroft's reading in aid of hospitals, has been written by Mr. Harry Ebdon, of Wandsworth Common, a humorous lecturer and author of numerous rhymes and readings. The poem has been very favourably received and acknowledged by His Royal Highness the Prince of Wales, Field Marshal Viscount Wolseley, the Earl of Halsbury, the Very Rev. the Chief Rabbi, and other eminent men.

The sub-committee appointed in 1897 to consider the question of expenditure at the Devon and Exeter Hospital have now presented their third report to the Special Committee of three which is considering the management of the hospital, and been approved by that committee. The recommendations include the appointment of a secretary at a salary of £150 per annum; that the office of house steward shall be abolished; that an assistant matron, at a salary of £40, rising to £50, be appointed; and also a dispenser, at a salary of £100 per annum.

The Canon in Residence, writing from the Manor House, Bristol, has made an analysis of the contributions of congregants at the cathedral at morning service on Hospital Sunday. There were between 800 and 900 persons (coming principally from the well-to-do and comfortable classes) attending, and the offertory was a little over £49. Of that sum about £33 was made up of a Bank of England note, two cheques, and gold, representing the contributions of some 50 persons. The 800 remaining accordingly subscribed about £16 between them, which gives an average contribution of 4½d. each.

### The Student of Medicine.

### APPOINTMENTS.

J. Anderson, M.A., M.B., C.M.Edin., B.Hy.Dunelm, appointed Medical Officer and Public Vaccinator for the Seaton Delaval District of the Tynemouth Union. R. J. Andrews, M.R.C.S.-Eng., appointed Medical Officer of Health to the Heavitree Urban District Council. J. H. Bellamy, L.R.C.P.Lond., M.R.C.S.Eng., appointed Junior Assistant Medical Officer to the Union Infirmary, Fir Vale, Sheffield. E. J. Cave, M.D.Lond., appointed Honorary Physician to the Royal United Hospital, Bath. F. H. Day, M.B., Ch.B.Vict., appointed House Surgeon to the Coventry and Warwickshire Hospital. W. Dunn, M.B., C.M.Aberd., appointed Medical Officer for the Mildenhall District of the Mildenhall Union. A. Heath, M.B., appointed Medical Officer for the Fifth District of the Aylsham Union. R. E. Howell, M.B, C.M.Edin., M.R.C.S.Eng., &c., appointed Honorary Surgeon to the North Ormesby Hospital, Middlesbrough-on-Tees. J. Miller, M.B, C.M.Aberd., appointed Medical Officer and Public Vaccinator for the Seghill District of the Tynemouth Union. F. B. Rutter, M.D.Durh., B.S., F.R.C.S., appointed Medical Officer for the Workhouse and the No. 1 District of the Mere Union. L. H. Walsh, M.B.Durh., appointed Honorary Assistant Physician to the Royal United Hospital, Bath. R. P. Woodroofe, L.R.C.S.I., L.A.H.Dub., appointed Medical Officer of Health to the Eccleshill Urban District Council. D. S. Wylie, M.B., Ch.B., appointed Junior Resident Medical Officer to the Manchester Children's Hospital.

